# Schein’s common sense emergency abdominal surgery (4th edition)

**DOI:** 10.1186/s13037-015-0088-z

**Published:** 2015-12-22

**Authors:** Philip F. Stahel

**Affiliations:** Department of Orthopaedics and Department of Neurosurgery, University of Colorado, School of Medicine Denver Health Medical Center, 777 Bannock Street, Denver, CO 80204 USA

“To cut or not to cut” is the ultimate challenge for any surgeon confronted with a patient who suffers from unclear abdominal symptoms. The decision of initiating appropriate and timely care with a surgical blade, weighed against the risk of delayed or negligent care by choosing observation or non-operative treatment reflects the eternal “moment of truth” for any surgeon on call. The current widespread fear of medicolegal litigation led to a modern practice of defensive medicine under which–as collateral damage–a surgeon’s experience and “common sense” approach has been all but extinguished. Who would be opposed to obtaining a CT scan for every unclear abdomen these days?

It’s time for a wake-up call. *Schein*’*s Common Sense* book series provides a refreshing, pragmatic and compelling advocacy for providing appropriate timely care to individual patients guided by practical tips and tricks, clinical decision-making, and sound judgment. The brand-new 4th edition of the *Emergency Abdominal Surgery* textbook (Box) brings Moshe Schein’s classic “Common Sense” series to a new pinnacle of quality and value for the reader. Building on the legacy of the three preceding editions, the new textbook expands into unchartered territory by emphasizing new concepts and modern developments, including an emphasis on the evolving role of laparoscopic approaches in the *Acute Care Surgery* paradigm.

**Book information** “Schein’s Common Sense: Emergency Abdominal Surgery“ (4th edition). Edited by Moshe Schein, Paul N. Rogers, Ari Leppaniemi, Danny Rosin, Jonathan E. Efron; TFM Publishing, Shropshire, UK, 2016. (ISBN: 978-1-910079-11-9)

The 4th edition has been significantly updated and features three new editors, additional authors, and new chapters. All existing chapters were extensively revised, expanded or rewritten. The main value of this encompassing textbook of 752 pages consists of the practical information conveyed to surgeons “in the trenches” by internationally renowned experts who have “seen it all and done it all.” The pragmatic approach of providing valuable technical tricks, decision-making algorithms and intraoperative bailout strategies is facilitated by the editors’ unwavering humorous style that has been established in the preceding editions as well as in other textbooks in the same series (e.g. *Schein*’*s Common SensePrevention and Management of Surgical Complications*, TFM, 2013, ISBN 978-1-903378-93-9).

Each chapter opens and ends with pertinent quotes, helpful mnemonics and suitable jokes that convey the content in a memorable fashion. The book’s “common sense” approach is amplified by the editors’ unique sense of humor that occasionally challenges our perception of political correctness (e.g. “*There are two things in life that we will never understand*: *women and acute appendicitis*.” *or* “*You can*’*t make chicken salad out of chicken shit*”). Impressively, the editors are able to walk the narrow margin of a sound sense of humor by continuously making fun of themselves, as portrayed in the opening cartoon that depicts the editors from an unfiltered human perspective (notably, with a bottle of milk, vodka, single malt whiskey and Italian wine on the editorial board desk). The need to have some fun to establish an honest and transparent educational approach is substantiated by the editors’ unquestionable credibility. The opening quote in the preface states suitably:

“Common sense and a sense of humor are the same thing, moving at different speeds. A sense of humor is just common sense, dancing. Those who lack humor are without judgment and should be trusted with nothing.”

The professional drawings that illustrate the textbook from A-Z have been comprehensively published in *The Little Book of Surgical Cartoons* (TFM, 2016, ISBN 978-1-910079-34-8). Clearly, these cartoons apply the final touch to a work that has been unequivocally cast in stone with the 4th edition as a “classic” among all available textbooks of surgery.

From a patient safety perspective, all 50 chapters focus on practical algorithms and technical tricks to keep our patients safe. In addition, common complications and respective bailout strategies are described in sufficient detail to allow even for junior surgeons on call to “get through the night” safely. Most importantly, this textbook allows surgeons to navigate the dangerous waters and temptation of providing unneeded surgery as one of the first and foremost root causes of preventable harm for our patients. In this regard, the final chapter on the “Aftermath and M&M meeting” provides the final authority from a patient safety perspective and strong imperative for open transparent reporting and analysis of all complications that occur under our watch, whether by errors in judgment or surgical technique, or both.

In summary, this exemplary textbook in its updated and expanded 4th edition represents an absolute “must” for the junior trauma and acute care surgeon on call, and an easy and entertaining read for any other adjunctive specialties (e.g. internal medicine or emergency medicine physicians) at risk of being confronted with the eternal conundrum in medicine: managing the patient with an acute and unclear abdomen.

